# The influence of fitness mobile apps on workout behavior intention among Chinese young adults

**DOI:** 10.1371/journal.pone.0320049

**Published:** 2025-03-27

**Authors:** Mengyu Li, Shujie Wang, Abdul Rahim Ahmed Soliman Darweesh

**Affiliations:** 1 School of Journalism and Communication, Henan University of Technology, Zhengzhou, China; 2 Academy of Arts and Communications, Qingdao Binhai University, Qingdao, China; 3 Radio & TV Department, Faculty of Mass Communication, Beni-Suef University, Beni Suef, Egypt; Universidade do Porto, PORTUGAL

## Abstract

Exercise was one of the most widely promoted methods to improve physical health while socially restricted. Despite the extensive notion of the advantages of sports activities, many young adults in China do not get the suggested amount of physical activity. Moreover, little is known about how one’s intentions regarding workout behavior are connected to one’s beliefs about using fitness mobile apps. The present study put into effect an online survey with random respondents of 5686 adults to examine a model combining the theory of planned behavior and the health belief model to predict Chinese adults’ attitudes, subjective norms, and perceived behavioral control when using fitness apps to work out, and to investigate associations between users’ beliefs and workout behavior intentions. The three main independent variables assessed were (1) attitude, (2) subjective norms, and (3) perceived behavioral control. Three components of belief were examined: self-efficiency, perceived barriers, and perceived benefits. The analyses found that attitudes and perceived behavioral control were positive and significant correlates of workout behavior intentions. The analysis also found that subjective norms were insignificantly associated with workout behavior intentions. Finally, the moderation test showed that belief moderated the assignations between the independent factors and workout behavior intentions. The findings suggest that the role of predictors within an integrated model using planned behavior theory and the health beliefs models provides a valid framework in the Chinese context of fitness app users. The research results have implications for the advancement and improvement of mobile phone apps that provide fitness functions, as well as for encouraging Chinese adults to promote the quantity and quality of their sport exercise.

## 1. Introduction

The World Health Organization (WHO) has been advancing global health since 1975. Since that time, surveys from WHO have shown that obesity case numbers have gone up almost threefold. Since obesity has got into one of the three most grievous health problems in China; furthermore, the number of obese people in the country is growing at the fastest rate in the world [1], and concerns among adult citizens about weight and body shape are continuing to rise [2]. With the remarkable evolution of advanced information and communication technology, digital technology has been gradually deployed in public health. Therefore, many researchers and practitioners have gone over to mobile health, also known as m-health [3]. As most mobile applications (apps) can be downloaded and accessed for free on smartphones, the opportunities for health-related services have increased. Moreover, the development and utilization of m-health apps for various purposes have grown significantly [4,5]. Furthermore, studies exploring m-health technologies have shown encouraging results, among which is that mobile fitness apps can enhance users to workout [6–8]. Fitness is an effective health intervention, and mobile apps have emerged to help people maintain health through fitness. They have happened quickly at an impressive speed in recent years [9,10]. These apps often offer users clues and advice on fitness classes, dietary advice, and weight loss tutorials [11,12]; moreover, they are considered to be low-cost and they make it feasible for busy, modern people to stay healthy. Finally, most m-health app users are trying to manage their diets and fitness routines rather than attempting to manage chronic illnesses or perform medical exams [13,14].

As other interventions have successfully used digital technology and big data to promote changes in health-related behaviors [15–17], m-health apps could serve as “catalysts” that remind people to stick with their fitness routines more consistently in order to obtain long-range health profits. Although the benefits of using m-health apps to increase physical activity and general wellness are widely known [18–20], it is still difficult for app system developers to create sustainable platforms for users, and for scholars to examine tactics to inspire people to continue using these platforms even after the originality has disappeared [21–23]. Nevertheless, although the significant outcomes associated with the widespread effectiveness of m-health apps for exercising and dieting [24–26], reserve rates for these apps remain low [27–29]. That is to say, users initially stay active, but quit after some time. Therefore, an urgent question is how to stimulate people’s fitness behavioral intentions continuously. Once this problem is overcome, m-health researchers and fitness app developers can design more user-friendly mobile applications that encourage users over the long term.

In China, big data on users’ personal information and needs is acquired and utilized for the development of mobile apps with various fitness-related functions; these apps have become an effective platform to encourage Chinese users to engage in physical activities [30,31]. Despite the utility and promise of such apps, the factors influencing users’ continuous usage intentions have not yet been examined. Based on the theory of planned behavior (TPB), the behavior of individuals who use the fitness functions offered by mobile apps is influenced by three key predictors, namely attitudes, subjective norms, and predictive behavioral control [32–36]. Furthermore, according to the health belief model (HBM), one way to support health is to maintain healthy beliefs [37–40], which consist of perceived susceptibility, perceived severity, perceived benefits, perceived barriers, and self-efficacy. These could ultimately change an individual’s behavioral intentions, and even help them achieve behavioral changes over the long term [41–43]. Therefore, by constantly stimulating users, fitness mobile applications could contribute to developing people’s quality of health behavior.

Although the accumulated findings provide insights for research on users of emerging fitness apps [44–46], there is a gap in the past research reflecting the scarcity of studies taking more sophisticated data to investigate variables that affect fitness behavioral intentions. Moreover, in China, this research is still in its elementary stages. To assess the correlation between the fitness behavior intentions, workout planning, and health beliefs of m-health app users, this study uses a comprehensive model that combines TPB and HBM to examine factors that influence individuals’ workout behavior intentions by analyzing data from mobile app users. The present research aims to empirically discuss adult users of five popular m-health apps in China: KEEP, Xiaomi Wear, Yue Dong Circle, Codoo, and Boohee. The following section presents a summary of these fitness mobile apps and a literature review related to the theoretical framework. Next, the methods used for data collection and analysis are described, and at last, the research results and recommendations are presented in detail.

## 2. Literature review

### 2.1. Fitness mobile apps in China

Fitness mobile apps can be broadly classified as functional technologies which aim to shift users’ attitudes or behaviors through persuasion and guidance rather than duress [4,10,47]. Several researchers have provided frameworks for understanding the adoption of fitness apps in China and their changing roles as tools, mediums, and social actors about health behavior [13,20,39]. First, as tools, fitness apps provide advice and data on peoples’ sports activity, such as dietary plans, steps taken, distance traveled, and calories ingested or burned [12,31,40]. This form of big data can present information on users’ exercise habits and physical changes, thus providing insight into their daily behavior and health [43,44,48]. Second, as mediums, fitness apps can serve as platforms for users to share their workout experiences and exchange information, motivating them to continue using the apps [25,49]. Third, as social actors, fitness apps can assist in implementing the “Healthy China Initiative” program (proposed by the Chinese government). They could prevail over users by approving them with significant replies and modeling test attitudes or behaviors related to a feasible workout [2,27,50]. In general, fitness apps provide information on health concepts and exercise trends in society.

This study focuses on fitness and health behavior changes among Chinese adults by exploring several of the most popular fitness apps in China. The contemporary problem of sub-optimal health has led to a strong awareness of health in society. People now pay more attention to workouts and fitness, and the development of the sports and physical industry is strongly promoted [31,39,51]. Regular fitness has become the new fashion for many young people [43,52,53]. “Fitness” generally refers to physical fitness, weight loss and toning, and fitness-related activities, such as running, swimming, yoga, and ball games. Fitness apps are divided into three main categories: (1) data recording applications, such as Xiaomi Wearer and Yue Dong Circle, which mainly record running times, routes, and calorie consumption; (2) fitness guidance applications, such as KEEP and Codoo, which allow users to self-customize their fitness training courses and are popular among most fitness enthusiasts; and (3) healthy diet guidance applications, such as Boohee, which provide tools like weight-loss recipes, fitness videos, and calorie counting and advocate healthy habits based on science. According to data from the Sutu Research Institute [54], 125.5 million Chinese adults used fitness app services in 2018. Since the influence of the global COVID-19 pandemic, people could not workout in gyms as usual, so KEEP, Xiaomi Wear, Yue Dong Circle, and Codoo all saw high numbers of downloads and active users. According to a report released by QuestMobile in February 2020, at that time, the number of active users of fitness apps had risen rapidly to 89.28 million in China [55]. Additionally, the number of monthly active users of fitness apps in April 2021 was more than 10 million [56]. Another popular fitness mobile app, Boohee, also had a large number of followers, with 4.17 million monthly active users in April 2021 [56]. This app is different from the four apps mentioned above, as it focuses on food, nutrition, and diet, providing users with health plan services.

As evidenced by the huge numbers of downloads and monthly active users, these fitness apps have several advantages. First, they are free; moreover, even though some fitness classes charge a fee, they are still affordable for most people, especially compared to the average gym membership [16,57]. Further, users can work out independently or receive appropriate guidance on how to exercise effectively [58,59]. Finally, users have been shown to engage in more physical activity after using a fitness app [30,60]. Similarly, Tu & Wang [53] reported on the positive effects of interventions on weight control among young people using technological means such as mobile apps. Research has also presented that the usage of fitness apps can improve users’ health awareness and ability to identify health risks [44,61,62]. In general, fitness apps can provide users with comprehensive health benefits [11,63,64].

### 2.2. Theoretical mechanisms

In order to explore the intentions of individuals using fitness apps, the study now turns to theoretical model construction. The current literature and the industry would both benefit from an in-depth analysis and understanding of fitness app users, and the following sections describe the theoretical perspectives on which this research is based. The theory of planned behavior (TPB) advises that the main parameter of behavior change is people’s intention to attract to the behavior [65–67]. According to this theoretical model, attitude, subjective norms, and perceived behavioral control jointly predict behavioral intentions [68–71]. Within the context of physical activity and fitness apps, attitude means the grade to which a user has a positive assessment of exercising. Subjective norms are beliefs about whether the important (reference) people in the user’s life approve or disapprove of their workout behavior, and the perceived social pressures to comply with those opinions. Perceived behavioral control refers to the extent to which a user believes people are capable of working out, and whether people perceive that they are in control of their workout or not.

The health belief model (HBM) is considered by many to be the leading cognitive model for the study of health behavior [72–75]. Especially, Janz & Becker’s HBM identified five original factors: (a) cues to action, (b) threat susceptibility, (c) threat severity, (d) barriers, and (e) benefits [76–81]. The decision to change one’s behavior depends upon the degree to which an individual (a) perceives a particular risk as directly threatening (perceived susceptibility +  perceived severity), (b) perceives their capability to execute a behavior required to produce specific outcomes (self-efficacy), and (c) believes that a specific action will prevent harm (response efficacy) [82–84]. Some scholars believe that health motivation is a significant concern to consider regarding health issues, so if good health is valued, health motivation becomes a key modifier in the HBM [85]. It has also served as a key organizing variable for health beliefs [86,87]. As a modifying factor, it has been presented to be positively associated with health consciousness, and behavioral intentions [88–90]. In this study, belief refers to a user’s perception of whether they will stick with exercise via a fitness app; more specifically, this includes the perceived benefits of using the fitness app; the perceived barriers to completing suggested actions, such as using a fitness app to work out or eat diet meals; and the perceptual capability to perform behaviors necessary to maintain health (self-efficacy) when using fitness applications.

Recent studies have demonstrated a better understanding of physical security behavior when pinpointing HBM variables are discussed as mediation variables [80,91]. Analogously, it is probable that all six factors are taken as equivalent mediators, demonstrating parallel mediation [92]; that some variables play a series mediating role, that is, they form sequential or serial chains [77]; or that the factors are hierarchically situated so that some moderate the meditation affect of others [91,93]. Therefore, an analysis of the effect of the HBM factors on the mediating variables and their contribution to preventive behavioral intentions may help to find chances for more successful communication channels. Moreover, these factors affecting behavioral intentions are the same in both the theory of planned behavior and the health belief model, and thus the role of beliefs could be added to the predicted findings of behavior health intentions, which evaluate the mediation effect between (1) attitudes, (2) subjective norms, and (3) perceived behavioral control and workout behavioral intentions.

**Fig 1:**
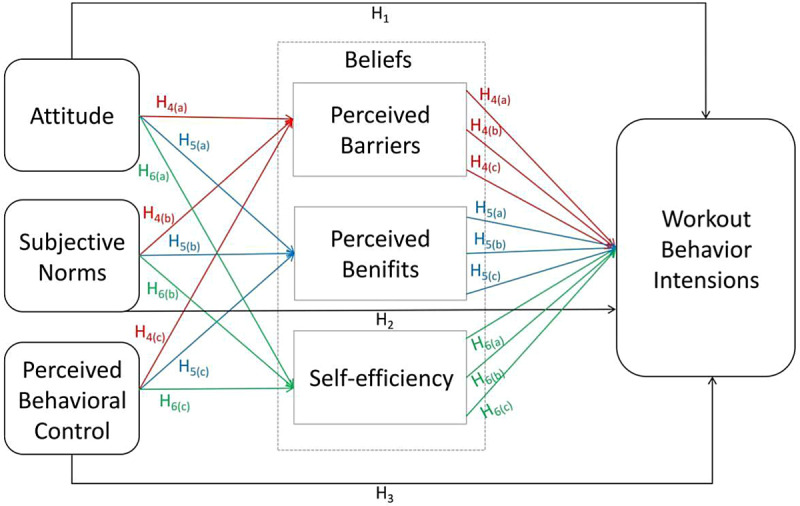
Theoretical model of independent variables, mediators, and dependent variable.

Scholars have answered to the growing fashion of fitness apps with a new wave of study exploring different sides of their use. This research is ongoing, and its targets continue to accumulate and diversify. However, three broad classifications of studies can be distinguished. The first set of research aims to check fitness app users, focusing on their evaluation of the apps and changes in their own physical activity [39,51,62–64]. The second set of studies explores the development and improvement of fitness apps to expand the usability and attractiveness of the software [13,22,24,29,57]. At last, a third set of research identifies a few predictors of fitness apps and online services [3,4,10,53]. These studies have mostly adopted the TPB, the HBM, the technology acceptance model (TAM) [94], and self-monitoring theory [95], along with theoretical models such as the unified theory of acceptance and use of technology (UTAUT) [96]. This present research has taken an optimized, comprehensive model using the TPB and the HBM to investigate the variables that affect individuals’ fitness behavior intentions. The theoretical framework proposed in this study to explore the associations between the factors is presented in Fig 1.

The study proposes the following hypotheses:

H_1_: Chinese adults’ attitudes towards using fitness apps will be positively associated with workout behavior intentions.

H_2_: Chinese adults’ subjective norms about using fitness apps will positively influence users’ workout behavior intentions.

H_3_: Chinese adults’ perceived behavior control regarding the use of fitness apps will positively predict their workout behavior intentions.

H_4(a)_: Perceived barriers will mediate the relationship between attitude and workout behavior intentions in Chinese adults using fitness apps.

H_4(b)_: Perceived benefits will play a mediating role between attitude and workout behavior intentions in Chinese adults using fitness apps.

H_4(c)_: The relationship between attitude and workout behavior intentions in Chinese adults using fitness apps will be mediated by self-efficacy.

H_5(a)_: Perceived barriers will mediate the relationship between subjective norms and workout behavior intentions in Chinese adults using fitness apps.

H_5(b)_: Perceived benefits will play a mediating role between subjective norms and workout behavior intentions in Chinese adults using fitness apps.

H_5(c)_: The relationship between subjective norms and workout behavior intentions in Chinese adults using fitness apps will be mediated by self-efficacy.

H_6(a)_: Perceived barriers will mediate the relationship between perceived behavior control and workout behavior intentions in Chinese adults using fitness apps.

H_6(b)_: Perceived benefits will play a mediating role between perceived behavior control and workout behavior intentions in Chinese adults using fitness apps.

H_6(c)_: The relationship between perceived behavior control and workout behavior intentions in Chinese adults using fitness apps will be mediated by self-efficacy.

## 3. Methods

This study conducted a questionnaire survey to examine the variables affecting the acceptance and usage of fitness apps. It did not specify a particular fitness app, as long as the participant was a user of one of the following five apps: KEEP, Xiaomi Wear, Yue Dong Circle, Codoo, or Boohee. Then, it showed the users how to make use of the functionality of these fitness applications to complete surveys and provide data. According to Ajzen’s [97] research, each variable should be defined within a specified frame and using a specific measurement. Throughout the study, all factors were measured on a five-point Likert scale ranging from 1 for “strongly disagree” to 5 for “strongly agree”.

The study recruited 5686 adults. The participants ranged in age from 18 to 65 years. Given the study’s focus on adults who use fitness apps, most data came from individuals at the lower end of the age range. After obtaining ethics approval from the participating university’s Human Research Ethics Committee (Academic Committee), participants were recruited through an online questionnaire link. The survey began from September 1, 2023 to October 31, 2023, during the period, the respondents can fill in the questionnaire online. Furthermore, all the respondents were informed of the ethics approval in detail, and they signed the consent forms before attending the study. They can quit the program anytime, and the study should keep their private information. Since the density of fitness app users in China is unevenly distributed, the data in the study were collected from online surveys. Moreover, the penetration rate of mobile devices is higher in first-tier cities, which have a wider and more accurate range of people who use fitness apps. Thus, the questionnaires were principally dispensed in the metropolises of big cities, such as Beijing, Shanghai, Guangzhou, and Shenzhen, four cities with similar populations. In each of the four cities, 1500 samples were distributed, for 6000 questionnaires distributed. The users then read an informed consent form explaining the research aims (i.e., completion of a short questionnaire) and the time required to complete the questionnaire (15–30 minutes). In the end, 5912 questionnaires were collected for a 98.5% recovery rate. Of these, 5686 were valid samples, for an effective rate of 96.2%, indicating the high validity of the questionnaires. Therefore, 5686 complete user profiles made up the final sample, and any cases that had missing data points or had been reused were eliminated as invalid samples.

Regarding the differences in generation densities, levels of economic development, and people’s awareness of fitness in various regions of China, the participants are chiefly from economically developed cities. The demographic characteristics of the respondents are shown in Table 1. Most of the samples are female (62.3%, n = 3542), and the proportion of males is 37.7% (n = 2144). From the perspective of age distribution, young people from 18 to 25 years of age make up the main of the respondents (39.8%, n = 2259), followed by those aged from 26 to 33 (33.2%, n = 1892). Referring to educational attainment, most users have a bachelor’s degree as their highest degree (41.3%, n = 2348). In the matter of employment status, the largest proportion of participants worked as corporate employees (45.8%, n = 2599), followed by students (31.1%, n = 1768).

**Table 1:** Demographic characteristics of the participants (n =  5686).

Type	Feature	N	%
Gender	Male	2144	37.7
	Female	3542	62.3
Age group	19–25	2259	39.8
	26–33	1892	33.2
	34–41	1049	18.4
	>42	486	8.2
Highest education level	Senior high school	1041	18.3
	Vocational school	1433	25.2
	Undergraduate	2348	41.3
	Postgraduate	864	15.2
Occupation category	Students	1768	31.1
	Corporate employees	2599	45.8
	Self-employed business owners	893	15.8
	Others	426	7.5

## 4. Results and discussion

### 4.1. Reliability and validity test

According to the recommendations of Gefen et al. [98], the data analysis was implemented in two steps. Firstly, the study assessed the reliability and two validity types: content and construct validity. In the large-sample analysis stage, the Cronbach’s alpha value and the Corrected Item-Total Correlation (CITC) were mainly used to measure the reliability of each index of the questionnaire. For the Cronbach’s alpha value, Wortzel [99] claimed that ≥0.6 is acceptable, and ≥0.7 indicates good reliability [100,101]. For the CITC, some studies have considered >  0.3 to be acceptable [102] and >  0.5 to be ideal [103]. In this study, the Cronbach’s alpha value of every variable is >  0.800, and the CITC value is >  0.6.

Second, the research ensured content validity by adopting measurement items from past studies. The study evaluated the content validity of the items themselves using the Kaiser-Meyer-Olkin (KMO) test and Bartlett’s test. Lao et al. [104] indicated that a KMO value ≥0.8 is suitable for factor analysis. Factor analysis is effective only when the p-value of Bartlett’s test is less than 0.05, and the closer it is to zero, the better the effect is [105]. In this study, factor analysis was conducted on the sample data from the variable scale to test the significance level of the KMO coefficient value and Bartlett sphericity. It was found that the KMO value of the variable is 0.817, and the p-value is less than 0.001, which portends good content validity.

However, the construct validity was evaluated mainly through confirmatory factor analysis, the degree of fit for the measurement items and the model was tested mainly using the chi-square to the degree of freedom ratio, Goodness of Fit Index (GFI), Normed Fit Index (NFI), Comparative Fit Index (CFI), and Root Mean Square Error of Approximation (RMSEA). In this study, principal component analysis was taken to extract the factors, and seven factors were extracted in total. The cumulative total explanatory variance is 73.303%, showing that the questionnaire has good structural validity and strong cumulative explanatory ability. The variance of common factors is between 0.500 and 0.900, implying that most of the variation of the variables investigated can be explained using the obtained common factors. Through the above analysis, and according to the item semantics and factor load size explored, 40 items were reserved and a seven-factor model was obtained.

Confirmatory factor analysis was conducted on the variable scale. The initial model has a good fitting result. The model was used to test the latent variables belonging to various intrinsic consistencies among the observed variables through confirmatory factor analysis. When these values are higher than the suggested cutoff values of 0.8, 0.6, and 0.8, individually, they show good construct reliability [106]. The combined reliabilities for the seven factors were 0.941, 0.901, 0.922, 0.894, 0.913, 0.892, and 0.918, which were all higher than 0.8. The Average Variance Extraction (AVE) values were 0.729, 0.646, 0.666, 0.629, 0.639, 0.625, and 0.618, thus supporting the convergent validity of constructs. Thus, the outcomes imply good construct validity.

### 4.3. Model and hypotheses testing

As shown in Table 2, after the MI correction, the fit indices of the model have improved to some extent. In addition, the fit indices of RMSEA, the Tucker–Lewis Index (TLI), the incremental fit index (IFI) and CFI reach the ideal standard. The value of CMIN/df is considerable but within an acceptable range due to the large sample size (N =  5686); moreover, Wheaton et al. [107] claimed this indicator can be ignored. Therefore, the model is a reasonable fit, since all of the indicators meet the standards set by Hair et al. [108].

**Table 2:** Modified model fitting result.

Indicator	CMIN/df	TLI	NFI	IFI	CFI	RMSEA
Analysis result	27.043	0.908	0.915	0.918	0.918	0.068
Ideal standard (good)	<3	>0.9	>0.9	>0.9	>0.9	<0.08
Ideal standard (acceptable)	3–5	0.7–0.9	0.7–0.9	0.7–0.9	0.7–0.9	0.08–1

The hypotheses were examined through Structural Equation Modeling (SEM) in Mplus. The simulated path-fitting results are presented in Table 3. The analysis reveals that the best performance of the model effectively supports the theoretical specifications, as most paths achieve statistical significance at the level of 0.05 or better, and the performance of the most significant path coefficients is as expected. Specifically, two paths in the main effects model show a positive significant effect. The p-values for “attitude and workout behavior intentions” and “perceived behavioral control and workout behavior intentions” are all less than 0.001, while their standard path coefficients are 0.112 and 0.143, respectively, indicating that attitude has a positive and significant effect on workout behavior intentions. This result confirms hypothesis H1. Moreover, perceived behavioral control has a significant and positive effect on workout behavior intentions, supporting hypothesis H3. However, the p-value for “subjective norms and workout behavior intentions” is 0.698, which is higher than 0.05, indicating that subjective norms have a negative and insignificant effect on workout behavior intentions; thus, hypothesis H2 is not supported. Among the effects of each independent variable on workout behavior intentions, the degree of effect of the attribute is expressed by the path coefficient in the consequent order: perceived behavioral control (0.143)>  attitude (0.112). Among the effects on intention to exercise, perceived behavioral control has a higher degree of influence, while attitude has a lower degree of influence.

**Table 3:** Main effects model path fitting outcome.

Path			Standard path coefficient	S.E.	C.R.	P-value
Workout behavior intentions	<---	Attitude	0.112	0.014	8.311	***
Workout behavior intentions	<---	Subjective norms	0.06	0.016	0.389	0.698
Workout behavior intentions	<---	Perceived behavioral control	0.143	0.016	10.659	***

### 4.4. Test for mediation variable

Mediating variable analyses were carried out using SEM with bootstrapping methods [109]. In this research, an SEM analysis was accomplished through Amos, with attitude as the independent factor and workout behavior intention as the dependent factor. Specifically, c denotes the total effect, a and b stand for effect size, a * b stands for mediation effect, and a * b stands for (95% BootCI). According to the findings, the mediating role of perceived barriers, perceived benefits, and self-efficiency are decomposed, and the outcomes are revealed in Table 4.

**Table 4:** Findings for the mediation model test.

Items	c	a	b	a * b	a * b	Effect ratio	Test results
	Total effect			Mediation effect	95% BootCI		
Attitude **= > ** Perceived barriers **= > ** Workout behavior intentions	0.112	0.116	0.288	0.033	0.082 -0.148	29.464%	partial mediation
Attitude **= > ** Perceived benefits **= > ** Workout behavior intentions		0.181	0.182	0.033	0.148 -0.213	29.464%	partial mediation
Attitude **= > ** Self-efficiency **= > ** Workout behavior intentions		0.148	0.229	0.034	0.115 -0.182	30.357%	partial mediation
Subjective norms **= > ** Perceived barriers **= > ** Workout behavior intentions	0.006	0.257	0.288	0.074	0.223 -0.291	>1	full mediation
Subjective norms **= > ** Perceived benefits **= > ** Workout behavior intentions		0.164	0.182	0.030	0.129 -0.198	>1	full mediation
Subjective norms **= > ** Self-efficiency **= > ** Workout behavior intentions		0.303	0.229	0.069	0.267 -0.338	>1	full mediation
Perceived behavioral control **= > ** Perceived barriers **= > ** Workout behavior intentions	0.143	0.252	0.288	0.073	0.220 -0.281	51.049%	partial mediation
Perceived behavioral control **= > ** Perceived benefits **= > ** Workout behavior intentions		0.216	0.182	0.039	0.184 -0.248	27.273%	partial mediation
Perceived behavioral control **= > ** Self-efficiency **= > ** Workout behavior intentions		0.199	0.229	0.046	0.170 -0.225	23.116%	partial mediation

The total effect of attitude on workout behavior intentions is 0.112, which passes the significance test (P <  0.05). In particular, the coefficients of attitude between perceived barriers, perceived benefits, and self-efficiency towards workout behavior intentions are 0.116, 0.186, and 0.148, respectively, which pass the significance test (P <  0.05). In addition, the coefficients of perceived barriers, perceived benefits, and self-efficiency towards workout behavior intentions are 0.288, 0.182, and 0.229, respectively, which pass the significance test (P <  0.05). Therefore, in the bootstrap 95% confidence interval of the mediation effect, its upper and lower bounds do not contain 0, indicating that attitude has a mediation effect on the correlations between the independent factors and dependent variable and, more specifically, attitude partially mediated the associations between factors and workout behavior intentions.

The total impact of subjective norms on workout behavior intentions is 0.006, which passes the significance test (P <  0.05). In particular, the coefficients of subjective norms between perceived barriers, perceived benefits, and self-efficiency towards workout behavior intentions are 0.257, 0.164, and 0.303, respectively, which pass the significance test (P <  0.05). Furthermore, the coefficients of perceived barriers, perceived benefits, and self-efficiency towards workout behavior intentions are 0.288, 0.182, and 0.229, respectively, which pass the significance test (P <  0.05). In other words, in the bootstrap 95% confidence interval of the mediation effect, its upper and lower bounds do not contain 0, indicating that subjective norms can be a mediating variable and, more accurately, reflect a full mediation effect.

The impact of perceived behavioral control towards workout behavior intentions is 0.112, which passes the significance test (P <  0.05). In particular, the coefficients of perceived behavioral control between perceived barriers, perceived benefits, and self-efficiency towards workout behavior intentions are 0.252, 0.216, and 0.199, respectively, which pass the significance test (P <  0.05). Moreover, the coefficients of perceived barriers, perceived benefits, and self-efficiency towards workout behavior intentions are 0.288, 0.182, and 0.229, respectively, which pass the significance test (P <  0.05). Thus, in the bootstrap 95% confidence interval of the mediation effect, its upper and lower bounds do not contain 0, indicating that perceived behavioral control can mediate the association between independent variables and workout behavior intentions, and, more precisely, it plays a partial mediation role.

## 5. Conclusion

The research outcomes demonstrate that attitudes and perceived behavioral control are positive predictive factors of fitness behavioral intentions among Chinese adults when using fitness mobile apps. Thus, stimulating fitness app users’ attitudes and perceived behavioral control can heighten workout intention. Additionally, subjective norms were not found to be a factor of the dependent variable, which is a valid contribution to the comprehensive model. Furthermore, beliefs were found to play a mediating role on the correlations between the independent factors and dependent variables; specifically, perceived benefits, self-efficacy intensify fitness behavioral intentions, while perceived barriers hinder workout behavioral intentions.

The research makes the following main contributions. First, as a theoretical contribution, it provides a complex theoretical model for the variables that propel the genuine use of mobile fitness applications. Through a thorough literature review of digital health technologies, in particular a summary of studies related to fitness app usage among Chinese adults, this study identifies a critical gap related to workout behavioral intentions. This study presents a comprehensive model combining TPB and HBM to examine the use of fitness apps by Chinese adults. While many related studies have focused on a single dependent variable or considered a combination of multiple models, the theoretical model proposed in this study goes beyond traditional models. In addition, it offers belief as a mediating variable to test its effect. Overall, the study not only illuminates the relationship between behavioral intentions and attitudes, subjective norms, predicted behavioral control, but also provides a more nuanced comprehension of the mediating role played by predicted benefits, predicted barriers, and self-efficacy in the relationships between the factors and dependent variables. These findings further broaden the theoretical and practical knowledge of digital public health phenomena.

Secondly, the empirical research investigated the behavioral intentions of users of fitness apps from the perspective of attitudes, subjective norms, predictive behavioral control and beliefs. Specifically, it integrated HBM into the framework of TPB to construct a new theoretical framework that incorporates a clearer perspective on user intentions related to fitness behavior. Although both TPB and HBM are extensively used in health communication research, neither model has provided strong proof that it can predict human behavioral intentions and behaviors. Furthermore, no clear combination rule has been developed to explain the variable relationship between the original models. Therefore, this study expands the relevant literature on fitness mobile apps, especially according to the mediating variable of beliefs. Moreover, this new model can be generalized to additional fields within health communication research and identify the factors concerned with taking advantage of digital technology in reality. Finally, the study provides some practical information for fitness mobile app developers; the outcomes can enable them to improve these apps by more effectively stimulating user behavioral intent, attracting new user downloads, and ensuring active users’ continued usage and satisfaction. From the perspective of indirect influence, the popularity and acceptance of fitness apps among Chinese adults directly reflect the effect of the country’s efforts to encourage physical exercise. Thus, this research substantially contributes to the future realization of the goals of the “Healthy China Initiative.”

In conclusion, this research addresses the effect of beliefs (perceived benefits, perceived barriers, and self-efficacy) as a mediation variable between attitudes, subjective norms, and perceived behavioral control and workout behavioral intention among Chinese fitness mobile app users. It also offers a more comprehensive understanding of the TPB and HBM models. With the development of digital technologies, both communication researchers and practitioners could better comprehend and influence health-relevant behaviors by paying more attention to the characteristics and attributes of these technologies.

Although this research makes many contributions and has various applications, the limitations would also be recognized. At first, since the research collected data in the form of online questionnaires, most of the participants in the survey were younger adults, ignoring the existence of China’s elderly population (this elderly group is keen on square dancing, and professional square dancing apps are available for them to use), so data on diverse groups are required in order to understand better the wider species group population of fitness app users. Second, the research focused on fitness app users, but only a subset of these users—namely users of five specific fitness apps—participated in the survey. Different apps have different characteristics, and there may be contrasts between their active user groups. Finally, since fitness apps’ various functions play different roles, there are differences between member users and ordinary users, as well as between paid users and users of free versions. Given these limitations, workout behavioral intentions should be investigated based on the different types of users. The research findings are generalized, and future research would view the various trends and directions. Moreover, the extent to which intentions affect behavior related to fitness app use should be observed. However, these considerations would not influence the outcomes and conclusions of the research.

## Supporting information

S1 File Questionnaire.
(DOC)

## References

[1] Ekai Capital Limited. 2021 China Health Industry White Paper; 2021. Availabble from: http://www.ceccapitalgroup.com/index.php?a=lists&catid=28&industry=&service_type=&happened=

[2] Wu R, Wang Q. Fitness APP: development status, problems and countermeasures. J Shandong Sport Univ. 2015;31(4):18–22.

[3] Free C, Phillips G, Felix L, Galli L, Patel V, Edwards P. The effectiveness of M-health technologies for improving health and health services: a systematic review protocol. BMC Res Notes. 2010;3:250. doi: 10.1186/1756-0500-3-250 20925916 PMC2976743

[4] Cho J, Park D, Lee HE. Cognitive factors of using health apps: systematic analysis of relationships among health consciousness, health information orientation, eHealth literacy, and health app use efficacy. J Med Internet Res. 2014;16(5):e125. doi: 10.2196/jmir.3283 24824062 PMC4035139

[5] Robbins R, Krebs P, Rapoport DM, Jean-Louis G, Duncan DT. Examining Use of Mobile Phones for Sleep Tracking Among a National Sample in the USA. Health Commun. 2019;34(5):545–51. doi: 10.1080/10410236.2017.1422104 29334765

[6] Hebden L, Cook A, van der Ploeg HP, Allman-Farinelli M. Development of smartphone applications for nutrition and physical activity behavior change. JMIR Res Protoc. 2012;1(2):e9. doi: 10.2196/resprot.2205 23611892 PMC3626164

[7] Pourzanjani A, Quisel T, Foschini L. Adherent Use of Digital Health Trackers Is Associated with Weight Loss. PLoS One. 2016;11(4):e0152504. doi: 10.1371/journal.pone.0152504 27049859 PMC4822791

[8] Voth EC, Oelke ND, Jung ME. A Theory-Based Exercise App to Enhance Exercise Adherence: A Pilot Study. JMIR Mhealth Uhealth. 2016;4(2):e62. doi: 10.2196/mhealth.4997 27307134 PMC4936794

[9] Cho J, Lee HE, Quinlan M. Complementary relationships between traditional media and health apps among american college students. J Am Coll Health. 2015;63(4):248–57. doi: 10.1080/07448481.2015.1015025 25692247

[10] Huang G, Zhou E. Time to Work Out! Examining the Behavior Change Techniques and Relevant Theoretical Mechanisms that Predict the Popularity of Fitness Mobile Apps with Chinese-Language User Interfaces. Health Commun. 2019;34(12):1502–12. doi: 10.1080/10410236.2018.1500434 30040501

[11] McArthur LH, Riggs A, Uribe F, Spaulding TJ. Health Belief Model Offers Opportunities for Designing Weight Management Interventions for College Students. J Nutr Educ Behav. 2018;50(5):485–93. doi: 10.1016/j.jneb.2017.09.010 29097024

[12] Molina MD, Sundar SS. Can mobile apps motivate fitness tracking? A study of technological affordances and workout behaviors. Health Communication. 2018;23(10):909–55. doi: 10.1080/10410236.2018.1536961 30358424

[13] Fu Y. Embodied media practice of fitness app users and self-construction of body problems. Social Science Research. 2021;7(5):206–12.

[14] Lupton D. Apps as Artefacts: Towards a Critical Perspective on Mobile Health and Medical Apps. Societies. 2014;4(4):606–22. doi: 10.3390/soc4040606

[15] Gold J, Lim MSC, Hocking JS, Keogh LA, Spelman T, Hellard ME. Determining the impact of text messaging for sexual health promotion to young people. Sex Transm Dis. 2011;38(4):247–52. doi: 10.1097/OLQ.0b013e3181f68d7b 20966830

[16] Mou X, Wang Q. Fitness APP user experience current conditions and development suggestions based on word frequency analysis. Journal of Physical Education. 2020;27(2):64–9. doi: 10.16237/j.cnki.cn44-1404/g8.2020.02.011

[17] Strandbygaard U, Thomsen SF, Backer V. A daily SMS reminder increases adherence to asthma treatment: a three-month follow-up study. Respir Med. 2010;104(2):166–71. doi: 10.1016/j.rmed.2009.10.003 19854632

[18] Kirwan M, Duncan MJ, Vandelanotte C, Mummery WK. Design, development, and formative evaluation of a smartphone application for recording and monitoring physical activity levels: the 10,000 Steps “iStepLog”. Health Educ Behav. 2013;40(2):140–51. doi: 10.1177/1090198112449460 22984196

[19] Sporrel K, De Boer RDD, Wang S, Nibbeling N, Simons M, Deutekom M, et al. The design and development of a personalized leisure time physical activity application based on behavior change theories, end-user perceptions, and principles from empirical data mining. Front Public Health. 2021;8:528472. doi: 10.3389/fpubh.2020.528472 33604321 PMC7884923

[20] Tang Y, Guo Q. Can fitness app use promote exercise behavior? - Based on the mediating effect of exercise self-efficacy and the moderating effect of social support. Journal of Chengdu Sport University. 2021;47(5):113–8. doi: 10.15942/j.jcsu.2021.05.018

[21] Becker S, Miron-Shatz T, Schumacher N, Krocza J, Diamantidis C, Albrecht U-V. mHealth 2.0: Experiences, Possibilities, and Perspectives. JMIR Mhealth Uhealth. 2014;2(2):e24. doi: 10.2196/mhealth.3328 25099752 PMC4114478

[22] Xiao L. Design of mobile app system for exercise fitness evaluation. Microcomputer application. 2020;36(10):18–20.

[23] Zhang D, Adipat B. Challenges, Methodologies, and Issues in the Usability Testing of Mobile Applications. International Journal of Human-Computer Interaction. 2005;18(3):293–308. doi: 10.1207/s15327590ijhc1803_3

[24] Chatzipavlou IA, Christoforidou SA, Vlachopoulou M. A recommended guideline for the development of mHealth Apps. Mhealth. 2016;2 21. doi: 10.21037/mhealth.2016.05.01 28293597 PMC5344150

[25] Lee HE, Cho J. What Motivates Users to Continue Using Diet and Fitness Apps? Application of the Uses and Gratifications Approach. Health Commun. 2017;32(12):1445–53. doi: 10.1080/10410236.2016.1167998 27356103

[26] Zhang L, Jiao Y. The spatial turn and value reconstruction of sports industry development in the post-epidemic era: Analysis of sports industry development based on Covid-19. Sports & Science. 2020;41(03):63–8. doi: 10.13598/j.issn1004-4590.2020.03.003

[27] Cai J, Sun J. Transaction cost model of involvement adjustment: Research on participation willingness of fitness APP users. Journal of Sports and Science. 2020;41(3):86. doi: 10.13598/j.issn1004-4590.2020.03.012

[28] Cho J. The impact of post-adoption beliefs on the continued use of health apps. Int J Med Inform. 2016;87 75–83. doi: 10.1016/j.ijmedinf.2015.12.016 26806714

[29] Krebs P, Duncan DT. Health App Use Among US Mobile Phone Owners: A National Survey. JMIR Mhealth Uhealth. 2015;3(4):e101. doi: 10.2196/mhealth.4924 26537656 PMC4704953

[30] Ashraf RU, Hou F, Ahmad W. Understanding Continuance Intention to Use Social Media in China: The Roles of Personality Drivers, Hedonic Value, and Utilitarian Value. International Journal of Human–Computer Interaction. 2018;35(13):1216–28. doi: 10.1080/10447318.2018.1519145

[31] Cui H, Chen Q. Study on the willingness of continuous mobile fitness app users. Journal of Capital University of Physical Education and Sports. 2020;32(1):75–83. doi: 10.14036/j.cnki.cn11-4513.2020.01.013

[32] Ajzen I. Constructing a theory of planned behavior questionnaire. University of Massachusetts Amherst; 2002. Available from: https://people.umass.edu/~aizen/pdf/tpb.measurement.pdf

[33] Johnson-Young EA. Predicting Intentions to Breastfeed for Three Months, Six Months, and One Year Using the Theory of Planned Behavior and Body Satisfaction. Health Commun. 2019;34(7):789–800. doi: 10.1080/10410236.2018.1437523 29485299

[34] Fontana J, Cranmer GA, Ash E, Mazer JP, Denham BE. Parent-Child Communication regarding Sport-Related Concussion: An Application of the Theory of Planned Behavior. Health Commun. 2022;37(8):923–34. doi: 10.1080/10410236.2021.1876326 33487037

[35] Kreitzberg DStC, Dailey SL, Vogt TM, Robinson D, Zhu Y. What is Your Fitness Tracker Communicating?: Exploring Messages and Effects of Wearable Fitness Devices. Qualitative Research Reports in Communication. 2016;17(1):93–101. doi: 10.1080/17459435.2016.1220418

[36] Freberg K. Using the Theory of Planned Behavior to predict intention to comply with a food recall message. Health Commun. 2013;28(4):359–65. doi: 10.1080/10410236.2012.688657 22746283

[37] Ahadzadeh AS, Pahlevan Sharif S, Ong FS, Khong KW. Integrating health belief model and technology acceptance model: an investigation of health-related internet use. J Med Internet Res. 2015;17(2):e45. doi: 10.2196/jmir.3564 25700481 PMC4376166

[38] Dennison L, Morrison L, Conway G, Yardley L. Opportunities and challenges for smartphone applications in supporting health behavior change: qualitative study. J Med Internet Res. 2013;15(4):e86. doi: 10.2196/jmir.2583 23598614 PMC3636318

[39] Wei J, Vinnikova A, Lu L, Xu J. Understanding and Predicting the Adoption of Fitness Mobile Apps: Evidence from China. Health Commun. 2021;36(8):950–61. doi: 10.1080/10410236.2020.1724637 32041437

[40] Yoo S-W, Kim J, Lee Y. The Effect of Health Beliefs, Media Perceptions, and Communicative Behaviors on Health Behavioral Intention: An Integrated Health Campaign Model on Social Media. Health Commun. 2018;33(1):32–40. doi: 10.1080/10410236.2016.1242033 27858470

[41] Harvey-Berino J, Pintauro SJ, Gold EC. The feasibility of using Internet support for the maintenance of weight loss. Behav Modif. 2002;26(1):103–16. doi: 10.1177/0145445502026001006 11799651

[42] Tang J, Abraham C, Stamp E, Greaves C. How can weight-loss app designers’ best engage and support users? A qualitative investigation. Br J Health Psychol. 2015;20(1):151–71. doi: 10.1111/bjhp.12114 25130682

[43] Zhu Y, Dailey SL, Kreitzberg D, Bernhardt J. “Social Networkout”: Connecting Social Features of Wearable Fitness Trackers with Physical Exercise. J Health Commun. 2017;22(12):974–80. doi: 10.1080/10810730.2017.1382617 29173072

[44] Elevelt A, Höhne JK, Blom AG. Squats in Surveys: Investigating the Feasibility of, Compliance With, and Respondents’ Performance on Fitness Tasks in Self-Administered Smartphone Surveys Using Acceleration Data. Front Public Health. 2021;9 627509. doi: 10.3389/fpubh.2021.627509 34616703 PMC8488116

[45] Iqbal U, Ho C-H, Li Y-CJ, Nguyen P-A, Jian W-S, Wen H-C. The relationship between usage intention and adoption of electronic health records at primary care clinics. Comput Methods Programs Biomed. 2013;112(3):731–7. doi: 10.1016/j.cmpb.2013.09.001 24091088

[46] Zhao W, Sun X, Wu S, Wu S, Hu C, Huo H, et al. MaGA20ox2f, an OsSD1 homolog, regulates flowering time and fruit yield in banana. Mol Breed. 2025;45(1):12. doi: 10.1007/s11032-024-01523-3 39803631 PMC11717755

[47] Fogg BJ. Persuasive technology: Using computers to change what we think and do. Boston, MA: Morgan Kaufmann Publishers; 2003.

[48] Wang J. Research on media equivalence of sports and fitness APP. Media. 2019;19 50–2.

[49] Zhang M, Liao J. The influence of fitness app usage on users’ running intentions-from the perspective of planned behavior theory. Journalism and Communications Review. 2018;7(12):91–4.

[50] Wang W, Cui J, Xing J. Influencing factors and multi-configuration path of continuous use of sports fitness apps in post-epidemic era-based on qualitative comparative analysis. Journal of Physical Adult Education. 2021;37(5):55–63. doi: 10.16419/j.cnki.42-1684/g8.2021.05.009

[51] Wang Q. Socialization, identity and presence: A study on the motivation and behavior of sports and fitness app users. Modern Communication (Journal of Communication University of China). 2018;40(12):149–56.

[52] Chen P, Wang Y. The identity construction of urban youth under the background of media sports socialization-taking fitness apps as an example. New Media Research. 2019;5(18):10–1. doi: 10.16604/j.cnki.issn2096-0360.2019.18.004

[53] Tu J, Wang R. Between out of control and control: Daily health practice of young people with new technology embedded. China Youth Research. 2019;12 21–9. doi: 10.19633/j.cnki.11-2579/d.2019.0156

[54] Sutu Research Institute. Statistical analysis of downloads of fitness apps in China; 2019. Available from: http://www.sootoo.com/

[55] QuestMobile. Quest Mobile Sports Fashion Consumer Insights Report; 2020. Available from: https://www.questmobile.com.cn/

[56] Xia. Analysis of the current situation and trend of my country’s sports and fitness industry in 2020; 2021. Available from: https://www.huaon.com/channeltrend/721863.html

[57] Xu L. Why did fitness apps get started so quickly-talking about the success of KEEP App. Publishing Wide Angle. 2015;1:160–16. doi: 10.16491/j.cnki.cn45-1216/g2.2015.0939

[58] Wang L. Research on the influence of sports APP on public fitness. Sports Science and Technology Literature Bulletin. 2021;29(10):48–9. doi: 10.19379/j.cnki.issn.1005-0256.2021.10.017

[59] Wu B, Wang Y. Research on the influencing factors of continuous use of mobile fitness APPs. Soft Science. 2019;33(10):87–92. doi: 10.13956/j.ss.1001-8409.2019.10.15

[60] Sun J, Cai J, Li T. Research on the user’s willingness to use sports fitness APP based on the two-factor theory. Journal of Physical Education. 2019;26(5).

[61] Cui H. Research on the intention to use mobile fitness app-based on technology readiness and Technology Acceptance Model (TAM). China Sports Science and Technology. 2022;1(1):1–10. doi: 10.16470/j.csst.2020116

[62] Liu S, Rui D. Research on the influence mechanism of social media contact on users’ exercise norm perception and fitness intention. Journalism Review. 2021;6(6):53–64. doi: 10.16057/j.cnki.31-1171/g2.2021.06.006

[63] Bryant K. Health benefits to using a fitness app. Club Solutions Magazine. 2015. Available from: https://clubsolutionsmagazine.com/2015/10/health-benefits-to-using-a-fitness-app/

[64] Byun H, Chiu W, Bae J. Exploring the Adoption of Sports Brand Apps. International Journal of Asian Business and Information Management. 2018;9(1):52–65. doi: 10.4018/ijabim.2018010105

[65] Fishbein M, Ajzen I. Predicting and changing behavior: The reasoned action approach. New York, NY: Psychology Press, Taylor & Francis Group; 2010.

[66] Hussain SA, Alhabash S. Nostalgic Emotional Valence and Its Effects on Help-Seeking in Depression. An Application of the Theory of Planned Behavior. Health Commun. 2021;36(13):1731–42. doi: 10.1080/10410236.2020.1794549 32698622

[67] Zhang N, Campo S, Yang J, Janz KF, Snetselaar LG, Eckler P. Effects of Social Support About Physical Activity on Social Networking Sites: Applying the Theory of Planned Behavior. Health Commun. 2015;30(12):1277–85. doi: 10.1080/10410236.2014.940669 26086237

[68] Ajzen I. The theory of planned behavior. Organizational Behavior and Human Decision Processes. 1991;50(2):179–211. doi: 10.1016/0749-5978(91)90020-t

[69] Jiang F, Lu S, Hou Y, Yue X. Dialectical thinking and health behaviors: the effects of theory of planned behavior. Int J Psychol. 2013;48(3):206–14. doi: 10.1080/00207594.2012.656130 22506588

[70] Manning M. The effects of subjective norms on behaviour in the theory of planned behaviour: a meta-analysis. Br J Soc Psychol. 2009;48(Pt 4):649–705. doi: 10.1348/014466608X393136 19187572

[71] Palmer CL, Burwitz L, Dyer AN, Spray CM. Endurance training adherence in elite junior netball athletes: a test of the theory of planned behaviour and a revised theory of planned behaviour. J Sports Sci. 2005;23(3):277–88. doi: 10.1080/02640410410001730098 15966346

[72] Atkin C, Marshall A. Health communication. An integrated approach to communication theory and research. 1996 479–96.

[73] Griffin MJ. Health belief model, social support, and intention to screen for colorectal cancer in older African American men. Health Promotion & Education. 2012;51(1):12–22.

[74] Lau R, Kane R, Berry S, Ware J, Roy D. Channeling health: a review of the evaluation of televised health campaigns. Health Educ Q. 1980;7(1):56–89. doi: 10.1177/109019818000700105 7275637

[75] Shafer A, Kaufhold K, Luo Y. Applying the Health Belief Model and an Integrated Behavioral Model to Promote Breast Tissue Donation Among Asian Americans. Health Commun. 2018;33(7):833–41. doi: 10.1080/10410236.2017.1315678 28467235

[76] Bresnahan M, Lee SY, Smith SW, Shearman S, Nebashi R, Park CY, et al. A theory of planned behavior study of college students’ intention to register as organ donors in Japan, Korea, and the United States. Health Commun. 2007;21(3):201–11. doi: 10.1080/10410230701307436 17567252

[77] Janz NK, Becker MH. The Health Belief Model: a decade later. Health Educ Q. 1984;11(1):1–47. doi: 10.1177/109019818401100101 6392204

[78] Maiman LA, Becker MH. The Health Belief Model: Origins and Correlates in Psychological Theory. Health Education Monographs. 1974;2(4):336–53. doi: 10.1177/109019817400200404

[79] Rosenstock IM. The health belief model: Explaining health behavior through expectancies. In Glanz K., Lewis FM., & Rime BK, editors. Health behavior and health education. San Francisco, CA: Jossey-Bass Publishers; 1990. p. 39–62.

[80] Witte K, Stokols D, Ituarte P, Schneider M. Testing the Health Belief Model in a Field Study to Promote Bicycle Safety Helmets. Communication Research. 1993;20(4):564–86. doi: 10.1177/009365093020004004

[81] Yuen KF, Ma F, Wang X, Lee G. The role of trust in influencing consumers’ adoption of automated vehicles: An application of the health belief model. International Journal of Sustainable Transportation. 2020;15(11):837–49. doi: 10.1080/15568318.2020.1821416

[82] Carpenter CJ. A meta-analysis of the effectiveness of health belief model variables in predicting behavior. Health Commun. 2010;25(8):661–9. doi: 10.1080/10410236.2010.521906 21153982

[83] Dikareva A, Harvey WJ, Cicchillitti MA, Bartlett SJ, Andersen RE. Exploring Perceptions of Barriers, Facilitators, and Motivators to Physical Activity Among Female Bariatric Patients: Implications for Physical Activity Programming. Am J Health Promot. 2016;30(7):536–44. doi: 10.4278/ajhp.140609-QUAL-270 26559717

[84] Yarbrough SS, Braden CJ. Utility of health belief model as a guide for explaining or predicting breast cancer screening behaviours. J Adv Nurs. 2001;33(5):677–88. doi: 10.1046/j.1365-2648.2001.01699.x 11298205

[85] Kegeles SS. A field experimental attempt to change beliefs and behavior of women in an urban ghetto. J Health Soc Behav. 1969;10(2):115–24. doi: 10.2307/2948359 5791236

[86] Champion VL. Revised susceptibility, benefits, and barriers scale for mammography screening. Res Nurs Health. 1999;22(4):341–8. doi: 10.1002/(sici)1098-240x(199908)22:4<341::aid-nur8>3.0.co;2-p 10435551

[87] Rosenstock IM. Historical Origins of the Health Belief Model. Health Education Monographs. 1974;2(4):328–35. doi: 10.1177/109019817400200403 299611

[88] Champion V, Skinner CS, Menon U. Development of a self-efficacy scale for mammography. Res Nurs Health. 2005;28(4):329–36. doi: 10.1002/nur.20088 16028267

[89] Kaufert JM, Rabkin SW, Syrotuik J, Boyko E, Shane F. Health beliefs as predictors of success of alternate modalities of smoking cessation: results of a controlled trial. J Behav Med. 1986;9(5):475–89. doi: 10.1007/BF00845134 3795265

[90] Hayes D, Ross CE. Concern with appearance, health beliefs, and eating habits. J Health Soc Behav. 1987;28(2):120–30. doi: 10.2307/2137126 3611701

[91] Champion V, Skinner CS. The health belief model. In Glanz K., Rimer B., Viswanath K., editors. Health behavior and health education. 4th ed. San Francisco, CA: Jossey-Bass; 2008. p. 45–65.

[92] Champion VL, Monahan PO, Springston JK, Russell K, Zollinger TW, Saywell RM Jr, et al. Measuring mammography and breast cancer beliefs in African American women. J Health Psychol. 2008;13(6):827–37. doi: 10.1177/1359105308093867 18697896 PMC2902247

[93] Champion VL. Instrument development for health belief model constructs. ANS Adv Nurs Sci. 1984;6(3):73–85. doi: 10.1097/00012272-198404000-00011 6426380

[94] El-Wajeeh M, H. Galal-Edeen ProfG, Mokhtar DrH. Technology Acceptance Model for Mobile Health Systems. IOSRJMCA. 2014;1(1):21–33. doi: 10.9790/0050-0112133

[95] Gleeson-Kreig JM. Self-monitoring of physical activity: effects on self-efficacy and behavior in people with type 2 diabetes. Diabetes Educ. 2006;32(1):69–77. doi: 10.1177/0145721705284285 16439495

[96] Oliveira T, Faria M, Thomas MA, Popovič A. Extending the understanding of mobile banking adoption: When UTAUT meets TTF and ITM. International Journal of Information Management. 2014;34(5):689–703. doi: 10.1016/j.ijinfomgt.2014.06.004

[97] Ajzen I. Perceived Behavioral Control, Self‐Efficacy, Locus of Control, and the Theory of Planned Behavior1. J Applied Social Pyschol. 2002;32(4):665–83. doi: 10.1111/j.1559-1816.2002.tb00236.x

[98] Gefen D, Karahanna E, Straub D. W. Trust and TAM in online shopping: An integrated model. MIS Quarterly. 2003;27(1):51–90. doi: 10.2307/30036519

[99] Wortzel R. Multivariate analysis. California Management Review. 1979;41:125–43.

[100] Fornell C, Larcker DF. Evaluating Structural Equation Models with Unobservable Variables and Measurement Error. Journal of Marketing Research. 1981;18(1):39. doi: 10.2307/3151312

[101] Nunnally JC, Bernstein IH. The assessment of reliability. Psychometric Theory. 1994;3 248–92.

[102] Ding S, Zhou X. A new reliability estimate. Journal of Jiangxi Normal University (Natural Science Edition). 2002;3:222–4.

[103] Cronbach LJ. Coefficient Alpha and the Internal Structure of Tests. Psychometrika. 1951;16(3):297–334. doi: 10.1007/bf02310555

[104] Lao J, Gu X, Tang G. Investigation and research on sports health behavior of middle school students based on SEM model. Sports Science and Technology Literature Bulletin. 2022;30(1):167–71. doi: 10.19379/j.cnki.issn.1005-0256.2022.01.048

[105] Raykov T. A Method for Examining Stability in Reliability. Multivariate Behav Res. 2000;35(3):289–305. doi: 10.1207/S15327906MBR3503_01 26745333

[106] Chin WW. The partial least squares approach to structural equation modeling. In Marcoulides G., editor. Modern methods for business research. Mahwah, NJ: Lawrence Erlbaum; 1998. p. 295–336.

[107] Wheaton B, Muthen B, Alwin DF, Summers GF. Assessing Reliability and Stability in Panel Models. Sociological Methodology. 1977;8 84. doi: 10.2307/270754

[108] Hair JF, Ringle CM, Sarstedt M. PLS-SEM: Indeed a Silver Bullet. Journal of Marketing Theory and Practice. 2011;19(2):139–52. doi: 10.2753/mtp1069-6679190202

[109] Hayes AF. Introduction to mediation, moderation, and conditional process analysis. New York, NY: The Guilford Press; 2013.

