# Supplementary material for: The influence of fitness mobile apps on workout behavior intention among Chinese young adults
Source: PLoS One. 2025 Mar 27;20(3):e0320049. doi: 10.1371/journal.pone.0320049 (PMC11949350; doi:10.1371/journal.pone.0320049)
Supplement: S1 File — Questionnaire. (DOC) [file pone.0320049.s001.doc]

Questionnaire

Dear respondents:

# My name is Li Mengyu. Currently working at Henan University of Technology. In fulfillment of the research project, I am conducting a study on “The Influence of Fitness Mobile Apps on Workout Behavior Intention among Chinese Young Adults”. The main objective of this study is to examine a model combining the theory of planned behavior and the health belief model to predict Chinese adults’ attitudes, subjective norms, and perceived behavioral control when using fitness apps to work out, and to investigate associations between users’ beliefs and workout behavior intentions. Thus, you are required to answer questionnaire. The research has been approved by School of Journalism and Communication, Zhengzhou University on 02/03/2024 where I obtained my master degree. If you have any questions about your rights as a participant in this study, please contact Dr. Jinglei Li, School of Journalism and Communication, Zhengzhou University.

Your response is very essential for the success of this study. Therefore, I would appreciate your honest and truthful answer in completing this online questionnaire. There are 9 sections including 45 questions, it will take you 15 minutes to finish. Please be assured that all your responses will be strictly confidential and will only be used for research purposes. We do not require you to write your name on the questionnaire. Only collected responses will be analyzed, and not individually. Your participation in this study is voluntary. You have right to withdraw at any time without having to provide reasons. Furthermore, for participants who withdraw, the researcher will not use their data for the study. This research does not involve any compensation or medical care.

If you have any inquiries on the online questionnaire, please do not hesitate to contact me at (emma_limengyu@sina.com). Alternatively, you can equally contact the correspondence author, Dr. Shujie Wang, at (Shujiewang0108@163.com).

Best Regards.

**Section A. Demographic Information**

Please tick (“√”) the one that applies to you in this section.

1. Gender: (1) Male □ (2) Female □

2. Age: (1) 18-25 □ (2) 26-33 □ (3) 34-41 □ (4)42 and Above □

3. Education background: (1) Senior high school □ (2) Vocational school □

(3) Undergraduate □ (4) Postgraduate □

4. Occupation: (1) Students □ (2) Corporate employees □

(3)Self-employed business owners □ (4)Others □

**Section B. Fitness Mobile Apps Usage Patterns**

Please tick (“√”) the one that applies to you in this section.

1. How many times per month do you workout via fitness mobile apps?

(1) Once □ (2)Twice □ (3) Three times □ (4) Four times □

(5) Five times or more □

2. How many hours do you spend using fitness mobile apps each month?

(1) Below 1 hour □ (2)1-5 hours □ (3) 6-10hours □ (4) 11 hours and above □

3. How many fitness mobile apps have you used until now?

(1) Below 1 app □ (2) 2-3 apps □ (3) 4-5 apps □

(4) 6 apps and above □

4. Which fitness mobile app do you prefer to use?

(1) KEEP □ (2) Xiaomi Wear □ (3) Yue Dong Circle □ (4) Codoo □

(5) Others □

**Section C. Attitude**

1. Strongly Disagree 2. Disagree 3. Somewhat Agree 4. Agree 5. Strongly Agree

Please rate (tick “√” the one) your agreement based on audience cognitive needs in viewing Chinese documentary programs.

| **Attitude** | 1 | 2 | 3 | 4 | 5 |
| --- | --- | --- | --- | --- | --- |
| I select the workout information from fitness mobile apps for my health. |  |  |  |  |  |
| I seek workout information from fitness mobile apps to help me obtain positive value towards life. |  |  |  |  |  |
| I choose the episode of workout videos uploaded on fitness mobile apps that will help me to lose wight. |  |  |  |  |  |
| I make sure that the information I select from fitness mobile apps suits my workout plan. |  |  |  |  |  |
| The information through fitness mobile apps can assist me increase my workout behavior rules and health knowledge. |  |  |  |  |  |

**Section D. Subject Norms**

1. Strongly Disagree 2. Disagree 3. Somewhat Agree 4. Agree 5. Strongly Agree

Please rate (tick “√” the one) your agreement based on audience affective needs in viewing Chinese documentary programs.

| **Subject Norms** | 1 | 2 | 3 | 4 | 5 |
| --- | --- | --- | --- | --- | --- |
| I use fitness mobile apps because of suggestions by other users. |  |  |  |  |  |
| Most of my friends do not use fitness mobile apps so I do not use it because of them. |  |  |  |  |  |
| I regard as fitness mobile app as perceived social pressures when I do not workout anymore. |  |  |  |  |  |
| I can get emotional resonance and insisting on workout when I view the videos uploaded by fitness mobile app users. |  |  |  |  |  |

**Section E. Perceived Behavior Control**

1. Strongly Disagree 2. Disagree 3. Somewhat Agree 4. Agree 5. Strongly Agree

Please rate (tick “√” the one) your agreement based on audience tension-free needs in viewing Chinese documentary programs.

| **Perceived Behavior Control** | 1 | 2 | 3 | 4 | 5 |
| --- | --- | --- | --- | --- | --- |
| I enjoyed working out through fitness mobile apps because it is a source of learning lifestyle. |  |  |  |  |  |
| Using fitness mobile apps enables me to be more confident and health because I can control my workout behavior. |  |  |  |  |  |
| I perceive that I am not in control of my working out via fitness mobile apps because it spends time. |  |  |  |  |  |
| My working out behavior via fitness mobile apps is for pleasure seeking in a stressful situation. |  |  |  |  |  |

**Section F. Perceived Barriers**

1. Strongly Disagree 2. Disagree 3.Somewhat Agree 4. Agree 5. Strongly Agree

Please rate (tick “√” the one) your agreement based on public perceptions of Chinese documentary programs.

| **Perceived Barriers** | 1 | 2 | 3 | 4 | 5 |
| --- | --- | --- | --- | --- | --- |
| I believe that health motivation to increase workout behavior intentions but the fitness mobile apps are not essential for me. |  |  |  |  |  |
| The information and knowledge on fitness mobile apps are not good for solving health issues and I will not workout anymore. |  |  |  |  |  |
| Reviewing the suggestions and discussions on fitness mobile apps not allows me to learn more about working out. |  |  |  |  |  |
| I can perceive the severity and threatening for my health if I do not use fitness mobile apps accurately. |  |  |  |  |  |
| Fitness mobile apps can not improve my workout behavior intentions because I perceive the working out is too hard for me. |  |  |  |  |  |

**Section G. Perceived Benefits**

1. Strongly Disagree 2. Disagree 3. Somewhat Agree 4. Agree 5. Strongly Agree

Please rate (tick “√” the one) your agreement based on audience satisfaction in viewing Chinese documentary programs.

| **Perceived Benefits** | 1 | 2 | 3 | 4 | 5 |
| --- | --- | --- | --- | --- | --- |
| I can get the the positive effects of interventions on weight control through fitness mobile apps. |  |  |  |  |  |
| Usage of fitness apps can improve my health awareness and enhance my workout behavior intentions. |  |  |  |  |  |
| I believes that follow the suggestions from fitness mobile will prevent harm when I workout. |  |  |  |  |  |
| I will stick with exercise via a fitness mobile app to completing suggested actions and eating diet meals to keep a perfect figure. |  |  |  |  |  |
| I can improve ability to identify health risks while I use fitness mobile apps. |  |  |  |  |  |
| I believe fitness mobile apps can provide users with comprehensive health benefits and health knowledge. |  |  |  |  |  |

**Section H. Self-efficacy**

1. Strongly Disagree 2. Disagree 3. Somewhat Agree 4. Agree 5. Strongly Agree

Please rate (tick “√” the one) your agreement based on continuous viewing intentions of Chinese documentary programs.

| **Self-efficacy** | 1 | 2 | 3 | 4 | 5 |
| --- | --- | --- | --- | --- | --- |
| When I use fitness mobile apps I feel convinced because I can control my health. |  |  |  |  |  |
| I perceive my capability to execute workout behavior required to produce good outcomes for my body via fitness mobile apps. |  |  |  |  |  |
| I want to engage in more physical activity after using fitness mobile apps since it can improve my ability to control my body. |  |  |  |  |  |
| I can work out independently or receive appropriate guidance on how to exercise effectively from fitness mobile apps. |  |  |  |  |  |
| I am satisfied with my health consciousness gained through fitness mobile apps so that it catch my intention to workout. |  |  |  |  |  |
| I am sure I have perceptual capability to perform behaviors necessary to maintain health when using fitness mobile apps. |  |  |  |  |  |

**Section I. Workout Behavior Intentions**

1. Strongly Disagree 2. Disagree 3. Somewhat Agree 4. Agree 5. Strongly Agree

Please rate (tick “√” the one) your agreement based on continuous viewing intentions of Chinese documentary programs.

| **Workout Behavior Intentions** | 1 | 2 | 3 | 4 | 5 |
| --- | --- | --- | --- | --- | --- |
| When I re-use the fitness mobile apps I feel convinced because I can perceive the workout behavior control. |  |  |  |  |  |
| I want to gain information by continuous using fitness mobile apps to enhance my level of health and reduce social pressures. |  |  |  |  |  |
| I will continue to use fitness mobile apps because I can perceive the benefits while I use them. |  |  |  |  |  |
| Continuous using fitness mobile apps makes me feel healthy and I can overcome the barriers when I workout. |  |  |  |  |  |
| I am satisfied with the usage experience(with friends or family) of fitness mobile apps so that they catch my attention to workout. |  |  |  |  |  |
| It will be a pleasant response for me to continue using fitness mobile apps since I am self -discipline. |  |  |  |  |  |

Thanks for your time and effort towards the success of this study.
